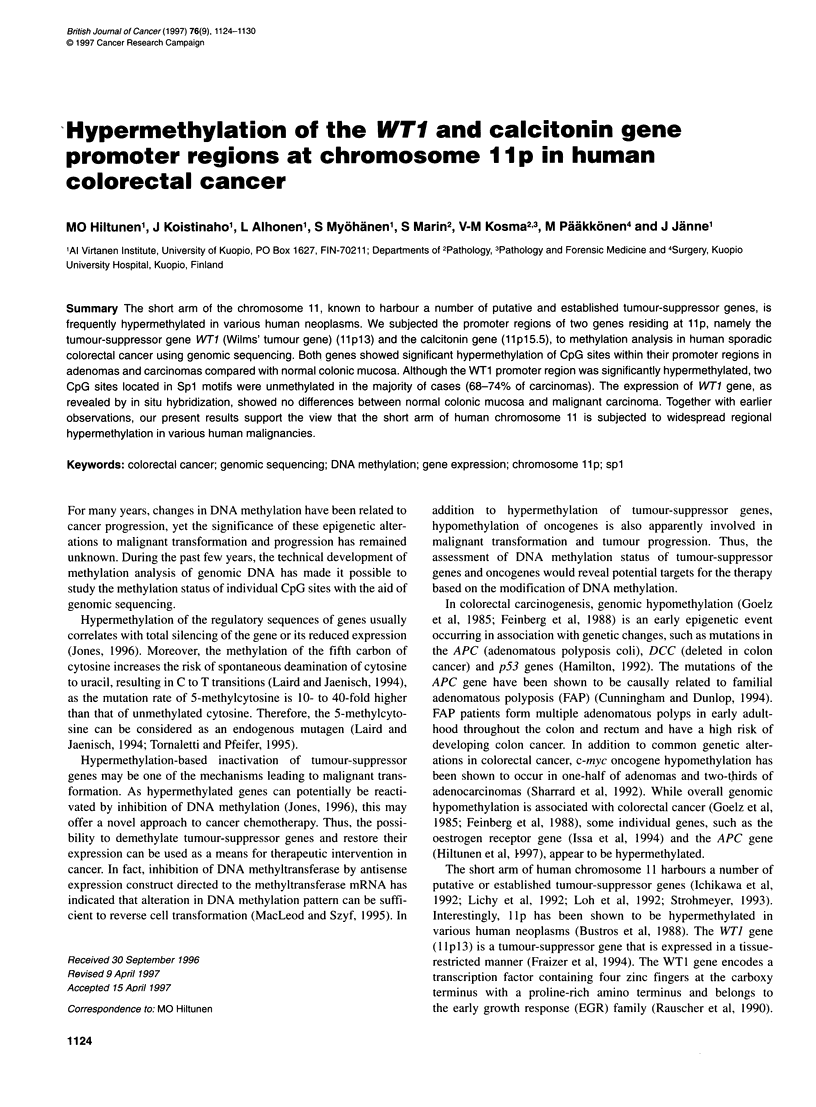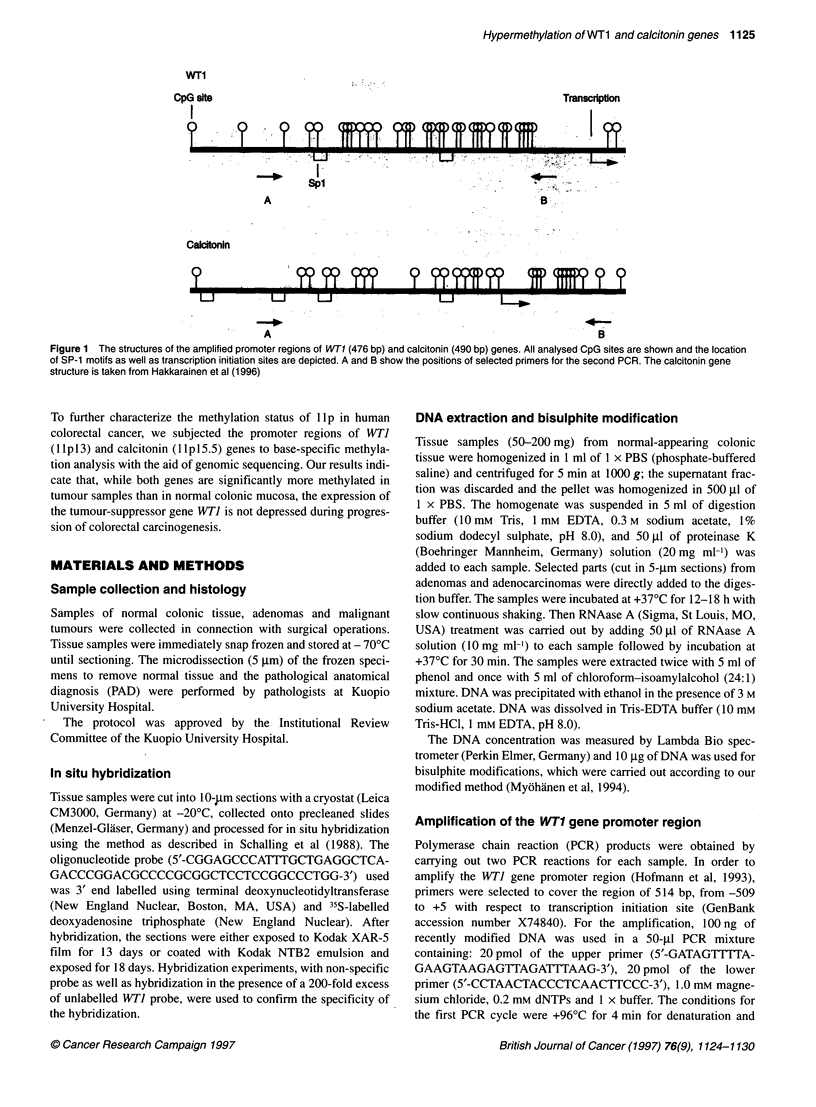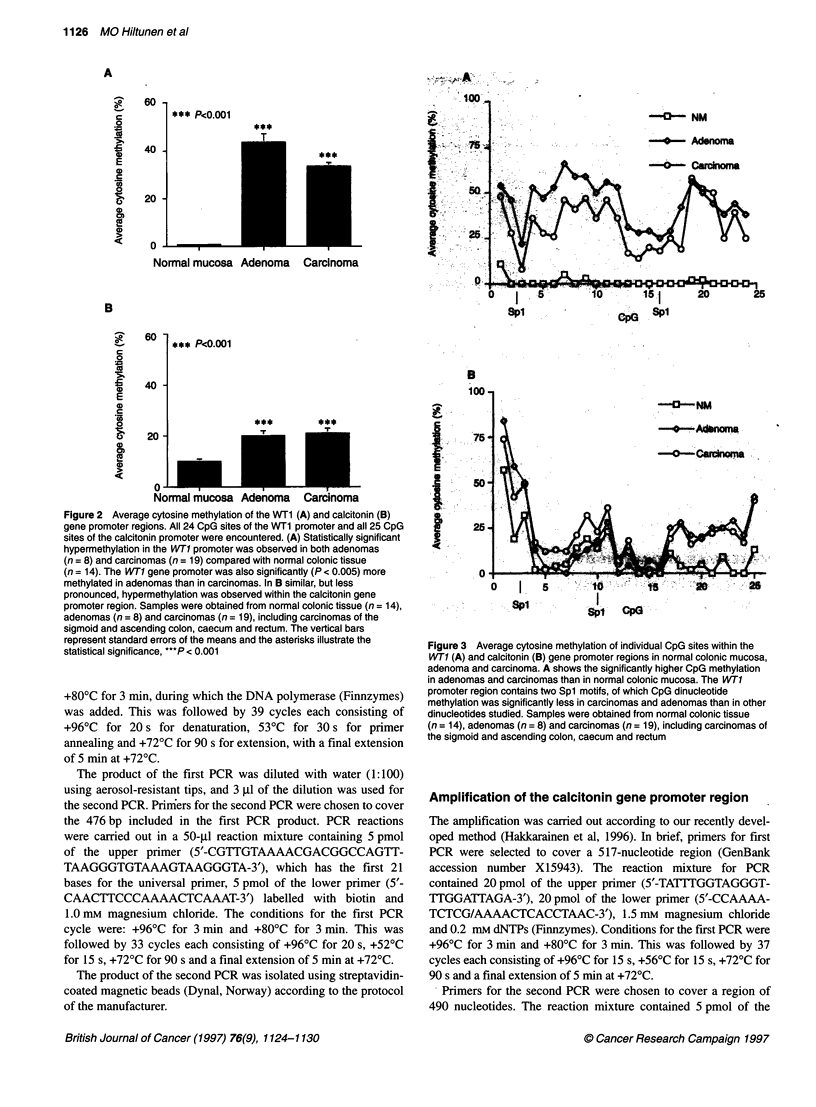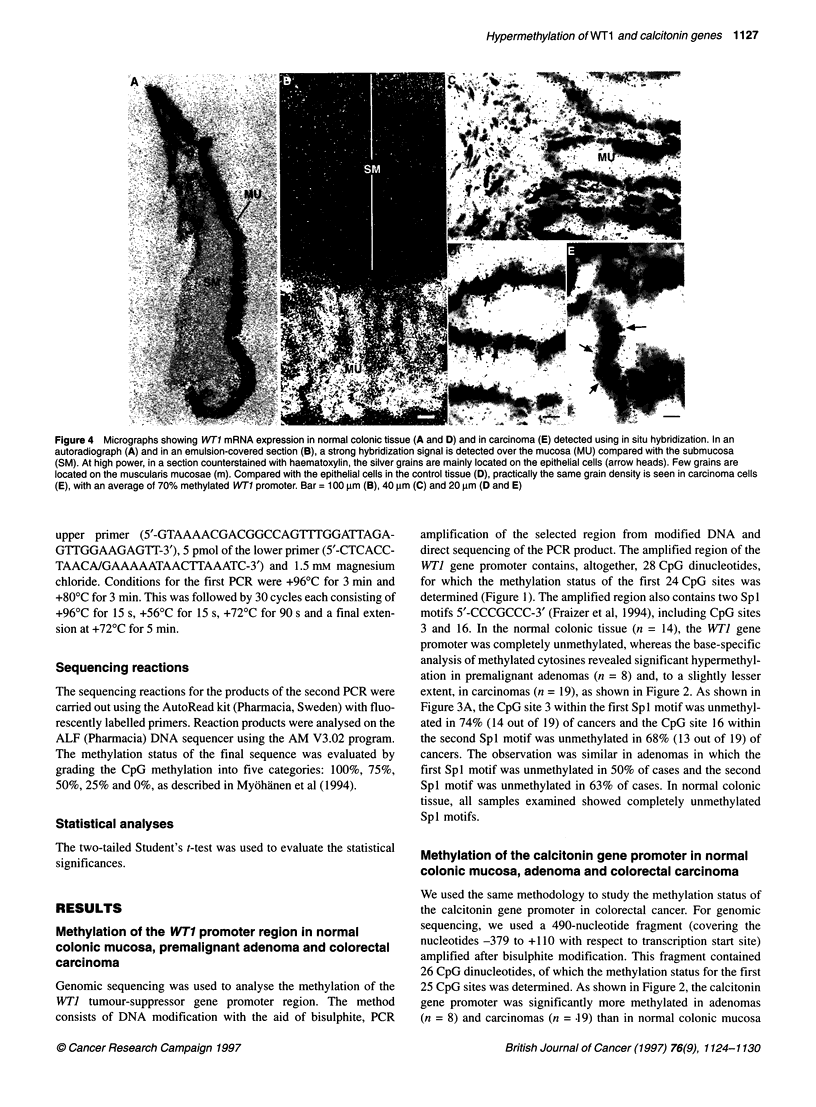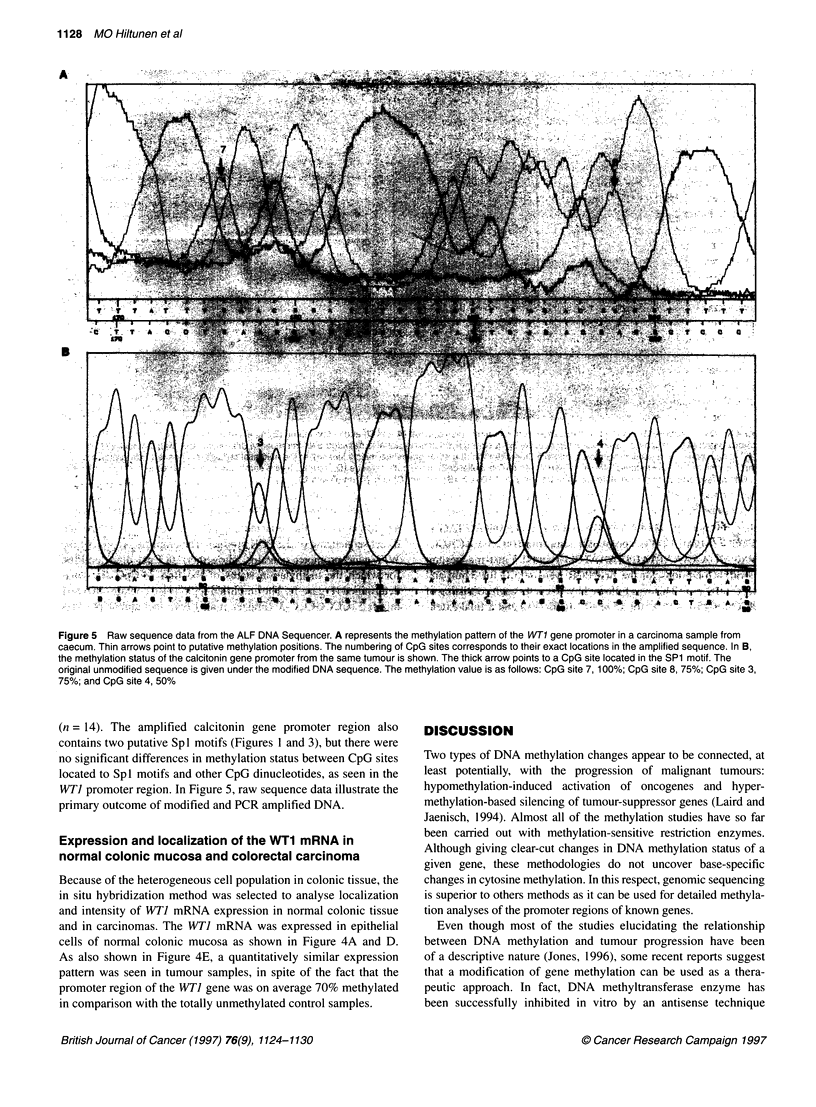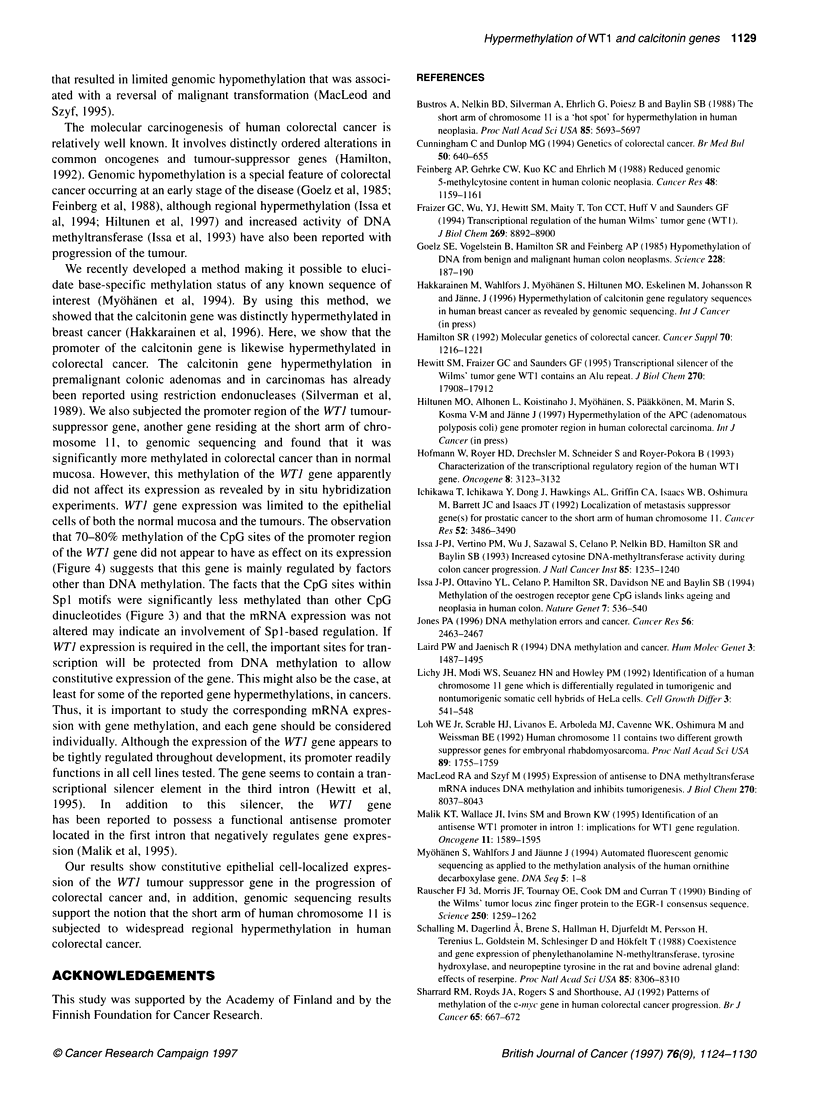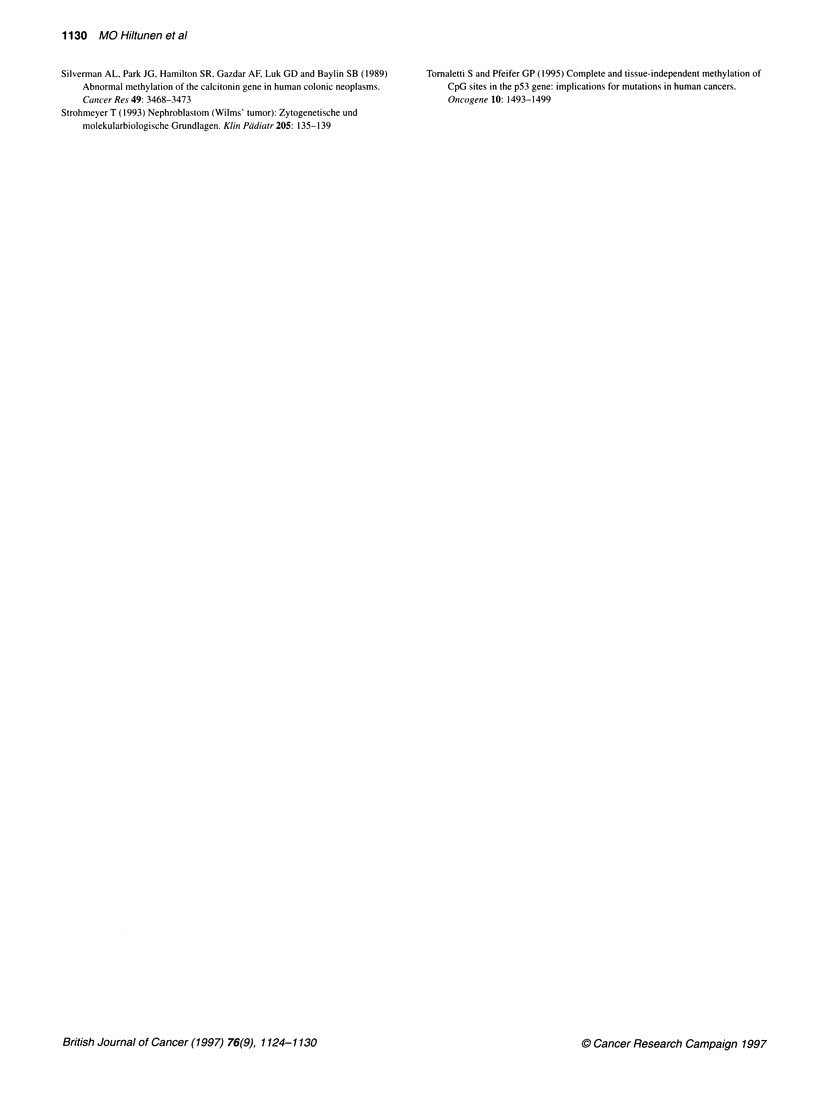# Hypermethylation of the WT1 and calcitonin gene promoter regions at chromosome 11p in human colorectal cancer.

**DOI:** 10.1038/bjc.1997.522

**Published:** 1997

**Authors:** M. O. Hiltunen, J. Koistinaho, L. Alhonen, S. MyÃ¶hÃ¤nen, S. Marin, V. M. Kosma, M. PÃ¤Ã¤kkÃ¶nen, J. JÃ¤nne

**Affiliations:** Al Virtanen Institute, University of Kuopio, Finland.

## Abstract

**Images:**


*British Journal of Cancer* (1997) **76**(9), 1124–1130
© 1997 Cancer Research Campaign

### Hypermethylation of the *WT1* and calcitonin gene promoter regions at chromosome 11p in human colorectal cancer

**MO Hiltunen[1], J Koistinaho[1], L Alhonen[1], S Myöhänen[1], S Marin[2], V-M Kosma[2,3], M Pääkkönen[4] and J Jänne[1]**

[1]AI Virtanen Institute, University of Kuopio, PO Box 1627, FIN-70211; Departments of [2]Pathology, [3]Pathology and Forensic Medicine and [4]Surgery, Kuopio University Hospital, Kuopio, Finland

**Summary** The short arm of the chromosome 11, known to harbour a number of putative and established tumour-suppressor genes, is frequently hypermethylated in various human neoplasms. We subjected the promoter regions of two genes residing at 11p, namely the tumour-suppressor gene *WT1* (Wilms' tumour gene) (11p13) and the calcitonin gene (11p15.5), to methylation analysis in human sporadic colorectal cancer using genomic sequencing. Both genes showed significant hypermethylation of CpG sites within their promoter regions in adenomas and carcinomas compared with normal colonic mucosa. Although the WT1 promoter region was significantly hypermethylated, two CpG sites located in Sp1 motifs were unmethylated in the majority of cases (68–74% of carcinomas). The expression of *WT1* gene, as revealed by in situ hybridization, showed no differences between normal colonic mucosa and malignant carcinoma. Together with earlier observations, our present results support the view that the short arm of human chromosome 11 is subjected to widespread regional hypermethylation in various human malignancies.

**Keywords:** colorectal cancer; genomic sequencing; DNA methylation; gene expression; chromosome 11p; sp1

For many years, changes in DNA methylation have been related to cancer progression, yet the significance of these epigenetic alterations to malignant transformation and progression has remained unknown. During the past few years, the technical development of methylation analysis of genomic DNA has made it possible to study the methylation status of individual CpG sites with the aid of genomic sequencing.

Hypermethylation of the regulatory sequences of genes usually correlates with total silencing of the gene or its reduced expression (Jones, 1996). Moreover, the methylation of the fifth carbon of cytosine increases the risk of spontaneous deamination of cytosine to uracil, resulting in C to T transitions (Laird and Jaenisch, 1994), as the mutation rate of 5-methylcytosine is 10- to 40-fold higher than that of unmethylated cytosine. Therefore, the 5-methylcytosine can be considered as an endogenous mutagen (Laird and Jaenisch, 1994; Tornaletti and Pfeifer, 1995).

Hypermethylation-based inactivation of tumour-suppressor genes may be one of the mechanisms leading to malignant transformation. As hypermethylated genes can potentially be reactivated by inhibition of DNA methylation (Jones, 1996), this may offer a novel approach to cancer chemotherapy. Thus, the possibility to demethylate tumour-suppressor genes and restore their expression can be used as a means for therapeutic intervention in cancer. In fact, inhibition of DNA methyltransferase by antisense expression construct directed to the methyltransferase mRNA has indicated that alteration in DNA methylation pattern can be sufficient to reverse cell transformation (MacLeod and Szyf, 1995). In addition to hypermethylation of tumour-suppressor genes, hypomethylation of oncogenes is also apparently involved in malignant transformation and tumour progression. Thus, the assessment of DNA methylation status of tumour-suppressor genes and oncogenes would reveal potential targets for the therapy based on the modification of DNA methylation.

In colorectal carcinogenesis, genomic hypomethylation (Goelz et al, 1985; Feinberg et al, 1988) is an early epigenetic event occurring in association with genetic changes, such as mutations in the *APC* (adenomatous polyposis coli), *DCC* (deleted in colon cancer) and *p53* genes (Hamilton, 1992). The mutations of the *APC* gene have been shown to be causally related to familial adenomatous polyposis (FAP) (Cunningham and Dunlop, 1994). FAP patients form multiple adenomatous polyps in early adulthood throughout the colon and rectum and have a high risk of developing colon cancer. In addition to common genetic alterations in colorectal cancer, c-*myc* oncogene hypomethylation has been shown to occur in one-half of adenomas and two-thirds of adenocarcinomas (Sharrard et al, 1992). While overall genomic hypomethylation is associated with colorectal cancer (Goelz et al, 1985; Feinberg et al, 1988), some individual genes, such as the oestrogen receptor gene (Issa et al, 1994) and the *APC* gene (Hiltunen et al, 1997), appear to be hypermethylated.

The short arm of human chromosome 11 harbours a number of putative or established tumour-suppressor genes (Ichikawa et al, 1992; Lichy et al, 1992; Loh et al, 1992; Strohmeyer, 1993). Interestingly, 11p has been shown to be hypermethylated in various human neoplasms (Bustros et al, 1988). The *WT1* gene (11p13) is a tumour-suppressor gene that is expressed in a tissue-restricted manner (Fraizer et al, 1994). The WT1 gene encodes a transcription factor containing four zinc fingers at the carboxy terminus with a proline-rich amino terminus and belongs to the early growth response (EGR) family (Rauscher et al, 1990).

*Received 30 September 1996*
*Revised 9 April 1997*
*Accepted 15 April 1997*

*Correspondence to:* MO Hiltunen

**Figure 1** The structures of the amplified promoter regions of *WT1* (476 bp) and calcitonin (490 bp) genes. All analysed CpG sites are shown and the location of SP-1 motifs as well as transcription initiation sites are depicted. A and B show the positions of selected primers for the second PCR. The calcitonin gene structure is taken from Hakkarainen et al (1996)

To further characterize the methylation status of 11p in human colorectal cancer, we subjected the promoter regions of *WT1* (11p13) and calcitonin (11p15.5) genes to base-specific methylation analysis with the aid of genomic sequencing. Our results indicate that, while both genes are significantly more methylated in tumour samples than in normal colonic mucosa, the expression of the tumour-suppressor gene *WT1* is not depressed during progression of colorectal carcinogenesis.

### MATERIALS AND METHODS

### Sample collection and histology

Samples of normal colonic tissue, adenomas and malignant tumours were collected in connection with surgical operations. Tissue samples were immediately snap frozen and stored at – 70°C until sectioning. The microdissection (5 µm) of the frozen specimens to remove normal tissue and the pathological anatomical diagnosis (PAD) were performed by pathologists at Kuopio University Hospital.

The protocol was approved by the Institutional Review Committee of the Kuopio University Hospital.

### In situ hybridization

Tissue samples were cut into 10-µm sections with a cryostat (Leica CM3000, Germany) at –20°C, collected onto precleaned slides (Menzel-Gläser, Germany) and processed for in situ hybridization using the method as described in Schalling et al (1988). The oligonucleotide probe (5′-CGGAGCCCATTTGCTGAGGCTCA-GACCCGGACGCCCCGCGGCTCCTCCGGCCCTGG-3′) used was 3′ end labelled using terminal deoxynucleotidyltransferase (New England Nuclear, Boston, MA, USA) and $^{35}$S-labelled deoxyadenosine triphosphate (New England Nuclear). After hybridization, the sections were either exposed to Kodak XAR-5 film for 13 days or coated with Kodak NTB2 emulsion and exposed for 18 days. Hybridization experiments, with non-specific probe as well as hybridization in the presence of a 200-fold excess of unlabelled *WT1* probe, were used to confirm the specificity of the hybridization.

### DNA extraction and bisulphite modification

Tissue samples (50–200 mg) from normal-appearing colonic tissue were homogenized in 1 ml of 1 × PBS (phosphate-buffered saline) and centrifuged for 5 min at 1000 ***g***; the supernatant fraction was discarded and the pellet was homogenized in 500 µl of 1 × PBS. The homogenate was suspended in 5 ml of digestion buffer (10 mM Tris, 1 mM EDTA, 0.3 M sodium acetate, 1% sodium dodecyl sulphate, pH 8.0), and 50 µl of proteinase K (Boehringer Mannheim, Germany) solution (20 mg ml$^{-1}$) was added to each sample. Selected parts (cut in 5-µm sections) from adenomas and adenocarcinomas were directly added to the digestion buffer. The samples were incubated at +37°C for 12–18 h with slow continuous shaking. Then RNAase A (Sigma, St Louis, MO, USA) treatment was carried out by adding 50 µl of RNAase A solution (10 mg ml$^{-1}$) to each sample followed by incubation at +37°C for 30 min. The samples were extracted twice with 5 ml of phenol and once with 5 ml of chloroform–isoamylalcohol (24:1) mixture. DNA was precipitated with ethanol in the presence of 3 M sodium acetate. DNA was dissolved in Tris-EDTA buffer (10 mM Tris-HCl, 1 mM EDTA, pH 8.0).

The DNA concentration was measured by Lambda Bio spectrometer (Perkin Elmer, Germany) and 10 µg of DNA was used for bisulphite modifications, which were carried out according to our modified method (Myöhänen et al, 1994).

### Amplification of the *WT1* gene promoter region

Polymerase chain reaction (PCR) products were obtained by carrying out two PCR reactions for each sample. In order to amplify the *WT1* gene promoter region (Hofmann et al, 1993), primers were selected to cover the region of 514 bp, from –509 to +5 with respect to transcription initiation site (GenBank accession number X74840). For the amplification, 100 ng of recently modified DNA was used in a 50-µl PCR mixture containing: 20 pmol of the upper primer (5′-GATAGTTTTA-GAAGTAAGAGTTAGATTTAAG-3′), 20 pmol of the lower primer (5′-CCTAACTACCCTCAACTTCCC-3′), 1.0 mM magnesium chloride, 0.2 mM dNTPs and 1 × buffer. The conditions for the first PCR cycle were +96°C for 4 min for denaturation and

© Cancer Research Campaign 1997

**Figure 2** Average cytosine methylation of the WT1 (**A**) and calcitonin (**B**) gene promoter regions. All 24 CpG sites of the WT1 promoter and all 25 CpG sites of the calcitonin promoter were encountered. (**A**) Statistically significant hypermethylation in the *WT1* promoter was observed in both adenomas ($n$ = 8) and carcinomas ($n$ = 19) compared with normal colonic tissue ($n$ = 14). The *WT1* gene promoter was also significantly ($P < 0.005$) more methylated in adenomas than in carcinomas. In **B** similar, but less pronounced, hypermethylation was observed within the calcitonin gene promoter region. Samples were obtained from normal colonic tissue ($n$ = 14), adenomas ($n$ = 8) and carcinomas ($n$ = 19), including carcinomas of the sigmoid and ascending colon, caecum and rectum. The vertical bars represent standard errors of the means and the asterisks illustrate the statistical significance, ***$P < 0.001$

**Figure 3** Average cytosine methylation of individual CpG sites within the *WT1* (**A**) and calcitonin (**B**) gene promoter regions in normal colonic mucosa, adenoma and carcinoma. **A** shows the significantly higher CpG methylation in adenomas and carcinomas than in normal colonic mucosa. The *WT1* promoter region contains two Sp1 motifs, of which CpG dinucleotide methylation was significantly less in carcinomas and adenomas than in other dinucleotides studied. Samples were obtained from normal colonic tissue ($n$ = 14), adenomas ($n$ = 8) and carcinomas ($n$ = 19), including carcinomas of the sigmoid and ascending colon, caecum and rectum

+80°C for 3 min, during which the DNA polymerase (Finnzymes) was added. This was followed by 39 cycles each consisting of +96°C for 20 s for denaturation, 53°C for 30 s for primer annealing and +72°C for 90 s for extension, with a final extension of 5 min at +72°C.

The product of the first PCR was diluted with water (1:100) using aerosol-resistant tips, and 3 µl of the dilution was used for the second PCR. Primers for the second PCR were chosen to cover the 476 bp included in the first PCR product. PCR reactions were carried out in a 50-µl reaction mixture containing 5 pmol of the upper primer (5′-CGTTGTAAAACGACGGCCAGTT-TAAGGGTGTAAAGTAAGGGTA-3′), which has the first 21 bases for the universal primer, 5 pmol of the lower primer (5′-CAACTTCCCAAAACTCAAAT-3′) labelled with biotin and 1.0 mM magnesium chloride. The conditions for the first PCR cycle were: +96°C for 3 min and +80°C for 3 min. This was followed by 33 cycles each consisting of +96°C for 20 s, +52°C for 15 s, +72°C for 90 s and a final extension of 5 min at +72°C.

The product of the second PCR was isolated using streptavidin-coated magnetic beads (Dynal, Norway) according to the protocol of the manufacturer.

### Amplification of the calcitonin gene promoter region

The amplification was carried out according to our recently developed method (Hakkarainen et al, 1996). In brief, primers for first PCR were selected to cover a 517-nucleotide region (GenBank accession number X15943). The reaction mixture for PCR contained 20 pmol of the upper primer (5′-TATTTGGTAGGGT-TTGGATTAGA-3′), 20 pmol of the lower primer (5′-CCAAAA-TCTCG/AAAACTCACCTAAC-3′), 1.5 mM magnesium chloride and 0.2 mM dNTPs (Finnzymes). Conditions for the first PCR were +96°C for 3 min and +80°C for 3 min. This was followed by 37 cycles each consisting of +96°C for 15 s, +56°C for 15 s, +72°C for 90 s and a final extension of 5 min at +72°C.

Primers for the second PCR were chosen to cover a region of 490 nucleotides. The reaction mixture contained 5 pmol of the

© Cancer Research Campaign 1997

**Figure 4** Micrographs showing *WT1* mRNA expression in normal colonic tissue (**A** and **D**) and in carcinoma (**E**) detected using in situ hybridization. In an autoradiograph (**A**) and in an emulsion-covered section (**B**), a strong hybridization signal is detected over the mucosa (MU) compared with the submucosa (SM). At high power, in a section counterstained with haematoxylin, the silver grains are mainly located on the epithelial cells (arrow heads). Few grains are located on the muscularis mucosae (m). Compared with the epithelial cells in the control tissue (**D**), practically the same grain density is seen in carcinoma cells (**E**), with an average of 70% methylated *WT1* promoter. Bar = 100 µm (**B**), 40 µm (**C**) and 20 µm (**D** and **E**)

upper primer (5′-GTAAAACGACGGCCAGTTTGGATTAGA-GTTGGAAGAGTT-3′), 5 pmol of the lower primer (5′-CTCACC-TAACA/GAAAAATAACTTAAATC-3′) and 1.5 mM magnesium chloride. Conditions for the first PCR were +96°C for 3 min and +80°C for 3 min. This was followed by 30 cycles each consisting of +96°C for 15 s, +56°C for 15 s, +72°C for 90 s and a final extension at +72°C for 5 min.

### Sequencing reactions

The sequencing reactions for the products of the second PCR were carried out using the AutoRead kit (Pharmacia, Sweden) with fluorescently labelled primers. Reaction products were analysed on the ALF (Pharmacia) DNA sequencer using the AM V3.02 program. The methylation status of the final sequence was evaluated by grading the CpG methylation into five categories: 100%, 75%, 50%, 25% and 0%, as described in Myöhänen et al (1994).

### Statistical analyses

The two-tailed Student's *t*-test was used to evaluate the statistical significances.

### RESULTS

### Methylation of the *WT1* promoter region in normal colonic mucosa, premalignant adenoma and colorectal carcinoma

Genomic sequencing was used to analyse the methylation of the *WT1* tumour-suppressor gene promoter region. The method consists of DNA modification with the aid of bisulphite, PCR amplification of the selected region from modified DNA and direct sequencing of the PCR product. The amplified region of the *WT1* gene promoter contains, altogether, 28 CpG dinucleotides, for which the methylation status of the first 24 CpG sites was determined (Figure 1). The amplified region also contains two Sp1 motifs 5′-CCCGCCC-3′ (Fraizer et al, 1994), including CpG sites 3 and 16. In the normal colonic tissue ($n$ = 14), the *WT1* gene promoter was completely unmethylated, whereas the base-specific analysis of methylated cytosines revealed significant hypermethylation in premalignant adenomas ($n$ = 8) and, to a slightly lesser extent, in carcinomas ($n$ = 19), as shown in Figure 2. As shown in Figure 3A, the CpG site 3 within the first Sp1 motif was unmethylated in 74% (14 out of 19) of cancers and the CpG site 16 within the second Sp1 motif was unmethylated in 68% (13 out of 19) of cancers. The observation was similar in adenomas in which the first Sp1 motif was unmethylated in 50% of cases and the second Sp1 motif was unmethylated in 63% of cases. In normal colonic tissue, all samples examined showed completely unmethylated Sp1 motifs.

### Methylation of the calcitonin gene promoter in normal colonic mucosa, adenoma and colorectal carcinoma

We used the same methodology to study the methylation status of the calcitonin gene promoter in colorectal cancer. For genomic sequencing, we used a 490-nucleotide fragment (covering the nucleotides –379 to +110 with respect to transcription start site) amplified after bisulphite modification. This fragment contained 26 CpG dinucleotides, of which the methylation status for the first 25 CpG sites was determined. As shown in Figure 2, the calcitonin gene promoter was significantly more methylated in adenomas ($n$ = 8) and carcinomas ($n$ = ·19) than in normal colonic mucosa

© Cancer Research Campaign 1997 

**Figure 5** Raw sequence data from the ALF DNA Sequencer. **A** represents the methylation pattern of the *WT1* gene promoter in a carcinoma sample from caecum. Thin arrows point to putative methylation positions. The numbering of CpG sites corresponds to their exact locations in the amplified sequence. In **B**, the methylation status of the calcitonin gene promoter from the same tumour is shown. The thick arrow points to a CpG site located in the SP1 motif. The original unmodified sequence is given under the modified DNA sequence. The methylation value is as follows: CpG site 7, 100%; CpG site 8, 75%; CpG site 3, 75%; and CpG site 4, 50%

($n = 14$). The amplified calcitonin gene promoter region also contains two putative Sp1 motifs (Figures 1 and 3), but there were no significant differences in methylation status between CpG sites located to Sp1 motifs and other CpG dinucleotides, as seen in the *WT1* promoter region. In Figure 5, raw sequence data illustrate the primary outcome of modified and PCR amplified DNA.

### Expression and localization of the WT1 mRNA in normal colonic mucosa and colorectal carcinoma

Because of the heterogeneous cell population in colonic tissue, the in situ hybridization method was selected to analyse localization and intensity of *WT1* mRNA expression in normal colonic tissue and in carcinomas. The *WT1* mRNA was expressed in epithelial cells of normal colonic mucosa as shown in Figure 4A and D. As also shown in Figure 4E, a quantitatively similar expression pattern was seen in tumour samples, in spite of the fact that the promoter region of the *WT1* gene was on average 70% methylated in comparison with the totally unmethylated control samples.

### DISCUSSION

Two types of DNA methylation changes appear to be connected, at least potentially, with the progression of malignant tumours: hypomethylation-induced activation of oncogenes and hypermethylation-based silencing of tumour-suppressor genes (Laird and Jaenisch, 1994). Almost all of the methylation studies have so far been carried out with methylation-sensitive restriction enzymes. Although giving clear-cut changes in DNA methylation status of a given gene, these methodologies do not uncover base-specific changes in cytosine methylation. In this respect, genomic sequencing is superior to others methods as it can be used for detailed methylation analyses of the promoter regions of known genes.

Even though most of the studies elucidating the relationship between DNA methylation and tumour progression have been of a descriptive nature (Jones, 1996), some recent reports suggest that a modification of gene methylation can be used as a therapeutic approach. In fact, DNA methyltransferase enzyme has been successfully inhibited in vitro by an antisense technique

 © Cancer Research Campaign 1997

that resulted in limited genomic hypomethylation that was associated with a reversal of malignant transformation (MacLeod and Szyf, 1995).

The molecular carcinogenesis of human colorectal cancer is relatively well known. It involves distinctly ordered alterations in common oncogenes and tumour-suppressor genes (Hamilton, 1992). Genomic hypomethylation is a special feature of colorectal cancer occurring at an early stage of the disease (Goelz et al, 1985; Feinberg et al, 1988), although regional hypermethylation (Issa et al, 1994; Hiltunen et al, 1997) and increased activity of DNA methyltransferase (Issa et al, 1993) have also been reported with progression of the tumour.

We recently developed a method making it possible to elucidate base-specific methylation status of any known sequence of interest (Myöhänen et al, 1994). By using this method, we showed that the calcitonin gene was distinctly hypermethylated in breast cancer (Hakkarainen et al, 1996). Here, we show that the promoter of the calcitonin gene is likewise hypermethylated in colorectal cancer. The calcitonin gene hypermethylation in premalignant colonic adenomas and in carcinomas has already been reported using restriction endonucleases (Silverman et al, 1989). We also subjected the promoter region of the *WT1* tumour-suppressor gene, another gene residing at the short arm of chromosome 11, to genomic sequencing and found that it was significantly more methylated in colorectal cancer than in normal mucosa. However, this methylation of the *WT1* gene apparently did not affect its expression as revealed by in situ hybridization experiments. *WT1* gene expression was limited to the epithelial cells of both the normal mucosa and the tumours. The observation that 70–80% methylation of the CpG sites of the promoter region of the *WT1* gene did not appear to have as effect on its expression (Figure 4) suggests that this gene is mainly regulated by factors other than DNA methylation. The facts that the CpG sites within Sp1 motifs were significantly less methylated than other CpG dinucleotides (Figure 3) and that the mRNA expression was not altered may indicate an involvement of Sp1-based regulation. If *WT1* expression is required in the cell, the important sites for transcription will be protected from DNA methylation to allow constitutive expression of the gene. This might also be the case, at least for some of the reported gene hypermethylations, in cancers. Thus, it is important to study the corresponding mRNA expression with gene methylation, and each gene should be considered individually. Although the expression of the *WT1* gene appears to be tightly regulated throughout development, its promoter readily functions in all cell lines tested. The gene seems to contain a transcriptional silencer element in the third intron (Hewitt et al, 1995). In addition to this silencer, the *WT1* gene has been reported to possess a functional antisense promoter located in the first intron that negatively regulates gene expression (Malik et al, 1995).

Our results show constitutive epithelial cell-localized expression of the *WT1* tumour suppressor gene in the progression of colorectal cancer and, in addition, genomic sequencing results support the notion that the short arm of human chromosome 11 is subjected to widespread regional hypermethylation in human colorectal cancer.

### ACKNOWLEDGEMENTS

This study was supported by the Academy of Finland and by the Finnish Foundation for Cancer Research.

### REFERENCES

Bustros A, Nelkin BD, Silverman A, Ehrlich G, Poiesz B and Baylin SB (1988) The short arm of chromosome 11 is a 'hot spot' for hypermethylation in human neoplasia. *Proc Natl Acad Sci USA* **85**: 5693–5697

Cunningham C and Dunlop MG (1994) Genetics of colorectal cancer. *Br Med Bul* **50**: 640–655

Feinberg AP, Gehrke CW, Kuo KC and Ehrlich M (1988) Reduced genomic 5-methylcytosine content in human colonic neoplasia. *Cancer Res* **48**: 1159–1161

Fraizer GC, Wu, YJ, Hewitt SM, Maity T, Ton CCT, Huff V and Saunders GF (1994) Transcriptional regulation of the human Wilms' tumor gene (WT1). *J Biol Chem* **269**: 8892–8900

Goelz SE, Vogelstein B, Hamilton SR and Feinberg AP (1985) Hypomethylation of DNA from benign and malignant human colon neoplasms. *Science* **228**: 187–190

Hakkarainen M, Wahlfors J, Myöhänen S, Hiltunen MO, Eskelinen M, Johansson R and Jänne, J (1996) Hypermethylation of calcitonin gene regulatory sequences in human breast cancer as revealed by genomic sequencing. *Int J Cancer* (in press)

Hamilton SR (1992) Molecular genetics of colorectal cancer. *Cancer Suppl* **70**: 1216–1221

Hewitt SM, Fraizer GC and Saunders GF (1995) Transcriptional silencer of the Wilms' tumor gene WT1 contains an Alu repeat. *J Biol Chem* **270**: 17908–17912

Hiltunen MO, Alhonen L, Koistinaho J, Myöhänen, S, Pääkkönen, M, Marin S, Kosma V-M and Jänne J (1997) Hypermethylation of the APC (adenomatous polyposis coli) gene promoter region in human colorectal carcinoma. *Int J Cancer* (in press)

Hofmann W, Royer HD, Drechsler M, Schneider S and Royer-Pokora B (1993) Characterization of the transcriptional regulatory region of the human WT1 gene. *Oncogene* **8**: 3123–3132

Ichikawa T, Ichikawa Y, Dong J, Hawkings AL, Griffin CA, Isaacs WB, Oshimura M, Barrett JC and Isaacs JT (1992) Localization of metastasis suppressor gene(s) for prostatic cancer to the short arm of human chromosome 11. *Cancer Res* **52**: 3486–3490

Issa J-PJ, Vertino PM, Wu J, Sazawal S, Celano P, Nelkin BD, Hamilton SR and Baylin SB (1993) Increased cytosine DNA-methyltransferase activity during colon cancer progression. *J Natl Cancer Inst* **85**: 1235–1240

Issa J-PJ, Ottavino YL, Celano P, Hamilton SR, Davidson NE and Baylin SB (1994) Methylation of the oestrogen receptor gene CpG islands links ageing and neoplasia in human colon. *Nature Genet* **7**: 536–540

Jones PA (1996) DNA methylation errors and cancer. *Cancer Res* **56**: 2463–2467

Laird PW and Jaenisch R (1994) DNA methylation and cancer. *Hum Molec Genet* **3**: 1487–1495

Lichy JH, Modi WS, Seuanez HN and Howley PM (1992) Identification of a human chromosome 11 gene which is differentially regulated in tumorigenic and nontumorigenic somatic cell hybrids of HeLa cells. *Cell Growth Differ* **3**: 541–548

Loh WE Jr, Scrable HJ, Livanos E, Arboleda MJ, Cavenne WK, Oshimura M and Weissman BE (1992) Human chromosome 11 contains two different growth suppressor genes for embryonal rhabdomyosarcoma. *Proc Natl Acad Sci USA* **89**: 1755–1759

MacLeod RA and Szyf M (1995) Expression of antisense to DNA methyltransferase mRNA induces DNA methylation and inhibits tumorigenesis. *J Biol Chem* **270**: 8037–8043

Malik KT, Wallace JI, Ivins SM and Brown KW (1995) Identification of an antisense WT1 promoter in intron 1: implications for WT1 gene regulation. *Oncogene* **11**: 1589–1595

Myöhänen S, Wahlfors J and Jäunne J (1994) Automated fluorescent genomic sequencing as applied to the methylation analysis of the human ornithine decarboxylase gene. *DNA Seq* **5**: 1–8

Rauscher FJ 3d, Morris JF, Tournay OE, Cook DM and Curran T (1990) Binding of the Wilms' tumor locus zinc finger protein to the EGR-1 consensus sequence. *Science* **250**: 1259–1262

Schalling M, Dagerlind Å, Brene S, Hallman H, Djurfeldt M, Persson H, Terenius L, Goldstein M, Schlesinger D and Hökfelt T (1988) Coexistence and gene expression of phenylethanolamine N-methyltransferase, tyrosine hydroxylase, and neuropeptine tyrosine in the rat and bovine adrenal gland: effects of reserpine. *Proc Natl Acad Sci USA* **85**: 8306–8310

Sharrard RM, Royds JA, Rogers S and Shorthouse, AJ (1992) Patterns of methylation of the c-*myc* gene in human colorectal cancer progression. *Br J Cancer* **65**: 667–672

© Cancer Research Campaign 1997

Silverman AL, Park JG, Hamilton SR, Gazdar AF, Luk GD and Baylin SB (1989) Abnormal methylation of the calcitonin gene in human colonic neoplasms. *Cancer Res* **49**: 3468–3473

Strohmeyer T (1993) Nephroblastom (Wilms' tumor): Zytogenetische und molekularbiologische Grundlagen. *Klin Pädiatr* **205**: 135–139

Tornaletti S and Pfeifer GP (1995) Complete and tissue-independent methylation of CpG sites in the p53 gene: implications for mutations in human cancers. *Oncogene* **10**: 1493–1499

*© Cancer Research Campaign 1997*